# Cryptopleurine Analogs with Modification of E Ring Exhibit Different Mechanism to Rac-Cryptopleurine and Tylophorine

**DOI:** 10.1371/journal.pone.0051138

**Published:** 2012-12-10

**Authors:** Ying Wang, Hui-Chyn Wong, Elizabeth A. Gullen, Wing Lam, Xiaoming Yang, Qian Shi, Kuo-Hsiung Lee, Yung-Chi Cheng

**Affiliations:** 1 Department of Pharmacology, Yale University School of Medicine, New Haven, Connecticut, United States of America; 2 Natural Products Research Laboratories, Eshelman School of Pharmacy, University of North Carolina, Chapel Hill, North Carolina, United States of America; 3 Chinese Medicine Research and Development Center, China Medical University and Hospital, Taichung, Taiwan; Ben-Gurion University of the Negev, United States of America

## Abstract

Tylophorine analogs exhibit a broad range of pharmacological activities, including anti-cancer, anti-inflammatory, anti-autoimmune, and anti-virus effects. Structure-activity relationship study of different structure tylophorine analogs can provide further understanding of their biological activity. Modifications on the **E** ring of the quinolizidine moiety of cryptopleurine analogs changed the potency and the selective inhibitory effect on NF-κB, AP-1, and CRE signaling pathways. Functional cryptopleurine analogs showed potent inhibition of NF-κB signaling pathway in both HepG2 and HEK-293 cell lines. The **E** ring structure analogs also differed in suppression of protein translation, and expression of cyclin D1. Our results showed that DCB-3503 or Rac-cryptopleurine could be a scaffold for modification to yield compounds with different mechanisms of action.

## Introduction

**Figure 1:**
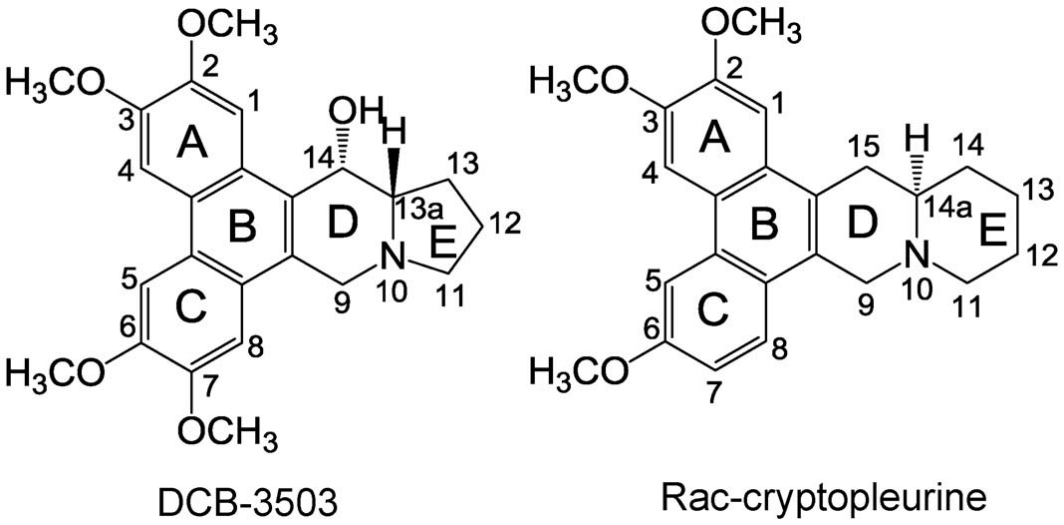
The chemical structures of DCB-3503 and Rac-cryptopleurine.

Tylophorine alkaloids are natural products originally identified in the *Asclepiadaceae* and *Moracea* family. Their claimed medical uses include the treatment of cancer, lupus, and inflammation [1], [2], [3], [4], [5], [6]. NCI’s COMPARE program indicated that their activity was distinct from other known anticancer compounds, suggesting that this group of analogs have a novel mode of action that is different from current chemotherapeutic compounds [1]. Tylophorine alkaloids, such as DCB-3500, DCB-3503, or Rac-cryptopleurine (chemical structures shown in Fig. 1), inhibit synthesis of protein, DNA, and RNA. DCB-3503 preferentially downregulated express of proteins with short a half-life, e.g. cyclin D1 [7], [8]. They exhibit inhibitory effect on NF-κB signaling pathway, but are less potent against activator protein-1 (AP-1), and cyclic AMP response elements (CREs) signaling pathways [1], [7], [9]. Tylophorine analog DCB-3503 but not DCB-3500 is active against HepG2 and PANC-1 xenografts in nude mice [1], [2], suggesting that the R14 hydroxyl group is important for *in vivo* activity.

Due to the diverse and potent pharmacological activities of tylophorine analogs, many groups including ours synthesize and modify different tylophorine alkaloids analogs and study their structure-activity relationship (SAR). This group of compounds shares a common pentacyclic structure with the phenanthrene ring conjugated with the indolizidine (five-member **E** ring) or quinolizidine (six-member **E** ring) moiety. Previous SAR study has found that cryptopleurine analogs with quinolizidine moiety (Fig. 1) were more active than analogs with indolizidine moiety against several cancer cell lines and NF-κB signaling pathway *in vitro* [9]. In the present study, we further evaluated SAR of a series of newly synthesized cryptopleurine analogs with modifications especially on the quinolizidine moiety.

## Results and Discussion

Our previous work demonstrated that Rac-cryptopleurine with the quinolizidine moiety (six-member **E** ring) is about 5 to 10 fold more potent than analogs with the indolizidine moiety (five-member **E** ring) (DCB-3500 and DCB-3503) [9]. In the current study, we analyzed the SAR of eleven cryptopleurine analogs with modifications especially on the quinolizidine moiety. These cryptopleurine analogs were previously reported by Dr. KH Lee’s laboratory [10], [11]. The hydroxylated analogs (YXM-109, -110, -139, and -140) are recently synthesized [12].

**Table 1:** The IC_50_ of tylophora alkaloids analogs on the growth inhibition of HepG2 and Huh 7 cells.

	HepG2 IC_50_^a^ (nM)	Huh-7 IC_50_^a^ (nM)
DCB-3503	91.0±11.2 ^b^	91.7±28.9
Rac-cryptopleurine	6.3±1.2 ^b^	2.0±0.1
YXM-109	134.0±28.0	42.0±32.0
YXM-110	49.0±15.0	5.0±4.1
YXM-140	258.0±70.0	21.3±8.8
	**HepG2 IC_50_^a^ (µM)**	**Huh-7 IC_50_^a^ (µM)**
YXM-66	1.4±0.2	1.1±0.1
YXM-82	1.1±0.2	1.0±0.2
YXM-83	2.7±0.2	0.3±0.2
YXM-93	0.82±0.13	1.2±0.2
YXM-101	2.4±0.4	4.0±0.3
YXM-139	1.6±0.2	3.75±0.3
YXM-142	4.0±0.2	1.0±0.2

The IC_50_ of these compounds are significantly different (*p*<0.05) from each other in different magnitude (nM vs µM); while compounds with the same magnitude (nM vs nM and µM vs µM) are not significantly different from each other (ANOVA analysis). ^a^ Values are means ± SD of at least three experiments, with each data point done in triplicate.^ b^ published.

The cytotoxicity of these compounds against human hepatoma cell lines (HepG2 and Huh-7) is shown in Table 1 (The dose-cell viability curves used to obtain the IC_50_ value is shown in Figure S1a and S1b). These compounds showed different selectivity against HepG2 and Huh-7 cell lines, suggesting that the mechanisms of action of different analogs could be cell-type specific. Structurally, YXM-109, 110, 139, and 140 differ only in the chirality and position of hydroxyl (-OH) group. The addition of a (R)-hydroxyl group on the R13 position (YXM-110) decreased the cytotoxicity to about seven fold in HepG2 cells and three fold in Huh-7 cells compared to Rac-cryptopleurine. The addition of an (S)-hydroxyl group on the R13 position (YXM-109) further decreased the cytotoxicity in both cells lines. YXM-140 with an (R)-hydroxyl group on the R12 position showed similar cytotoxic potencies compared to YXM-109. However, the addition of an (S)-hydroxyl group on the R12 position (YXM-139) resulted in loss of potency against the tested cancer cell lines. Modifications on the **E**-ring quinolizidine moiety, including substitution of carbon by nitrogen or oxygen (YXM-66, 82, 93, and 101), replacing the six-membered **E**-ring with a seven-membered **E**-ring (YXM-83) or with a five-membered N-cyclopropylpyrrolidinyl **E**-ring (YXM-93), caused the loss of cytotoxicity towards HepG2 and Huh-7 cell lines. Together with our previous report, these newly synthesized compounds showed similar spectrum of potency against A549, DU145, KB, KBvin, SKBR3 cell lines to HepG2 and Huh 7 cell lines as we reported in the present study [12]. The more potent cryptopleurine analogs (Rac-cryptopleurine, YXM-109, YXM-110, and YXM-140) exhibited moderate selectivity (nM IC_50_) against KB, KBvin, and Huh 7 cell line. These results provided important information that the structure requirements for the most potent cytotoxic analogs for different cell lines appear to be the same.

NF-κB plays important role in controlling inflammation, cancer cell survival and death, and formation of chemoresistance [10], [11]. Regulation of transcription factors including AP-1 and CRE governs key steps in controlling cell proliferation, inflammation, and apoptosis [13], [14]. Those three signal transduction pathways also interplayed among themselves. Inhibition of NF-κB signaling pathway is involved in the inhibition of cancer cell growth and suppression of inflammatory diseases in DCB-3503-treated mice model [2], [5], [13], [15]. DCB-3503, Rac-cryptopleurine, and their functional analogs preferentially inhibited NF-κB to AP-1, and CRE signaling pathways in HepG2 cell line [1], [7], [9]; therefore, we determined the activities of these eleven new cryptopleurine analogs to the above three signaling pathways in HepG2 and HEK-293 cell lines (The dose-luciferase activity curves used to obtain the IC_50_ value is shown in Figure S2a-S3c for HepG2 cells and S3a-S3c for HEK-293 cells). Results in Table 2 obtained from HepG2 cells showed that DCB-3503 and Rac-cryptopleurine preferentially inhibited NF-κB signaling pathway. YXM-140 had similar pattern of selectivity. YXM-109 and 110 had almost equal potency against NF-κB and AP-1 signaling pathways. YXM-139 with a R12-(S)-OH substitution showed at least 1000-fold less potent than its R13 -OH isomers (YXM-109, -110) and R12 enantioisomer (YXM-140). YXM-142 exhibited about 3-fold more potent against AP-1 than against NF-κB signaling pathway. YXM-93 and -139 inhibited CRE pathway preferentially in HepG2 cells. While YXM-66, 82, 83, and 101 exhibited almost equal activities to the three tested signaling pathways in HepG2 cells. Comparatively in HEK-293 cell lines (Table 2), Rac-cryptopleurine, YXM-109, 110, 140, and 82 showed potent inhibition on both NF-κB and AP-1 signaling pathways. The selectivity of YXM-93 and YXM-66 changed to AP-1 pathway. YXM-139 was active against both AP-1 and CRE signaling pathway. Despite the switches of selectivity against different signaling pathways of those less potent cryptopleurine analogs (YXM-66, 82, 93, 101, 139, 142), the sensitivity of more potent analogs (DCB-3503, Rac-cryptopleruine, YXM-83, 142, and 101) against NF-κB signaling pathway did not change in both HepG2 and HEK-293 cell lines. This result suggests that inhibition of NF-κB rather than AP-1 and CRE signaling pathway could be one of the key factors related to the potency of cryptopleurine analogs.

**Table 2:** The IC_50_ of the inhibitory effect of tylophora alkaloids on NF-κB, AP-1, and CRE signalging pathways in HepG2 and HEK-293 cells.

	NF-κB IC_50_ (nM)		AP-1 IC_50_ (nM)		CRE IC_50_ (nM)	
	HepG2	HEK-293	HepG2	HEK-293	HepG2	HEK-293
DCB-3503	85.0±7.1 ^a^	37.6±19.7	1500±330 ^a^	125±2.5	1067±231 ^a^	1815±970
Rac-cryptopleurine	1.5±0.28 ^a^	7.1±1.3	15.0±0.14 ^a^	8.8±3.0	30.0±0.58 ^a^	24.2±10.6
YXM-109	10.0±0.11	22.2±9.6	20.0±0.17	18.6±1.1	3000±503	8450±2190
YXM-110	10.0±0.12	20.2±1.5	15.0±0.14	5.6±2.0	500±70	460±230
YXM-140	15.0±0.14	22.4±7.9	41.2±18.0	16.7±2.6	1500±120	2200±280
YXM-142	153.5±4.9	425±77	50.0±0.2	60.1±1.5	30.0±0.17**µM**	3.9±1.4** µM**
	**NF-κB IC_50_ (µM)**		**AP-1 IC_50_ (µM)**		**CRE IC_50_ (µM)**	
	**HepG2**	**HEK-293**	**HepG2**	**HEK-293**	**HepG2**	**HEK-293**
YXM-66	>30.0	33.5±13.4	>30.0	5.4±1.2	>30.0	>30.0
YXM-82	15.0±0.32	1.1±0.6	5.0±0.85	0.62±0.03	>30.0	>30.0
YXM-83	3.0±0.1	5.0±0.4	1.5±0.85	5.8±0.7	1.5±0.14	3.1±0.8
YXM-93	15.0±0.71	26.3±8.2	15.0±0.67	1.95±0.7	1.0±0.13	>30.0
YXM-101	>30.0	120.0±14.1	>30.0	30.0±0.1	>30.0	>30.0
YXM-139	>30.0	79.4±15.2	>30.0	12.0±3.9	3.0±0.64	7.3±2.4

Values are means ± SD of at least three experiments, with each data point done in triplicate. The IC_50_ concentrations of these compounds are significantly different (*p*<0.05) from each other in between nM and µM concentrations; while compounds with the same concentration level (nM vs nM and µM vs µM) are not significantly different (ANOVA analysis). ^a^ published.

**Figure 2:**
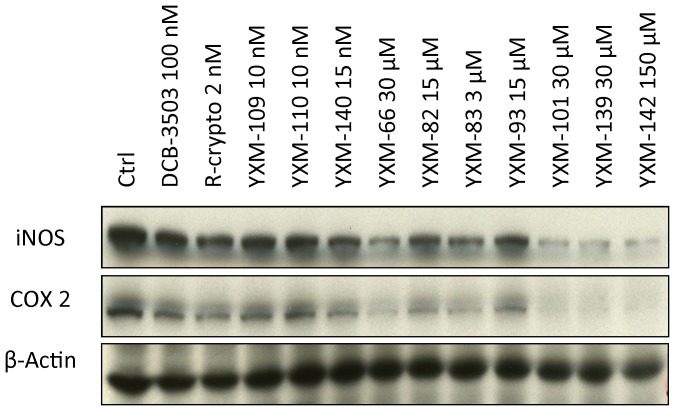
Crytopleurine analogs inhibited Cox 2 and iNOS expression. HepG2 cells were treated with crytopleurine analogs and DCB-3503 with the dosage as indicated in the figure for 4 hours. Protein level of Cox 2 and iNOS was analyzed by Western blot. Results were representative of three independent experiments.

Activation of NF-κB will induce the expression of Cox 2 [16] and iNOS [17], we then analyzed the effect of the treatment of cryptopleurine analogs on these two NF-κB pathway downstream targets by Western blot. Cryptopleurine analogs down-regulated the expression of both Cox 2 and iNOS in HepG2 cells at their IC_50_ concentration against NF-κB pathway (Fig. 2). This confirmed the inhibition of NF-κB pathway by cryptopleurine analogs. These results showed that modifications on the **E**-ring of cryptopleurine analogs are directly related to their selectivity against NF-κB, AP-1, and CRE signaling pathways in HepG2 cell line. Structural analogs can not only lead to altered potency [9], but also change mechanisms of action.

**Figure 3:**
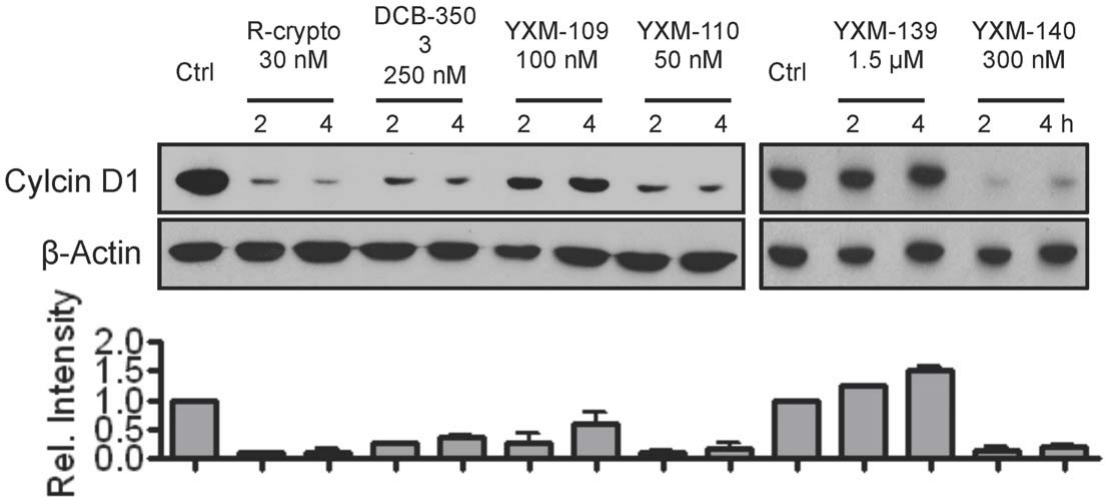
Functional crytopleurine analogs inhibited cyclin D1 expression. HepG2 cells were treated with crytopleurine analogs and DCB-3503 with the dosage as indicated in the figure for 2 and 4 hours. Cyclin D1 protein level was analyzed by Western blot. The band intensity of Cyclin D1 on Western blot was normalized with that of β-actin by densitometer scanning, and presented as a chart below. Results were mean ± SD from three independent experiments.

DCB-3503 suppressed the expression of cellular proteins with a short half-life, for instance cyclin D1 and p53 [8]. Therefore, we examined the effect of the treatment of functional crytopleurine analogs on the expression of cyclin D1. Figure 3 showed that the treatment of Rac-cryptopleurine, and DCB-3503 with about three times IC_50_ concentration decreased more than 70% of cyclin D1 expression. The treatment of YXM-109, -110, and -140 also showed more than 50% inhibitory effect on cyclin D1 expression at their IC_50_ concentration in HepG2 cells (Fig. 3). However, the treatment of YXM-139 did not change cyclin D1 expression level at its IC_50_ concentration (Fig. 3).

**Figure 4:**
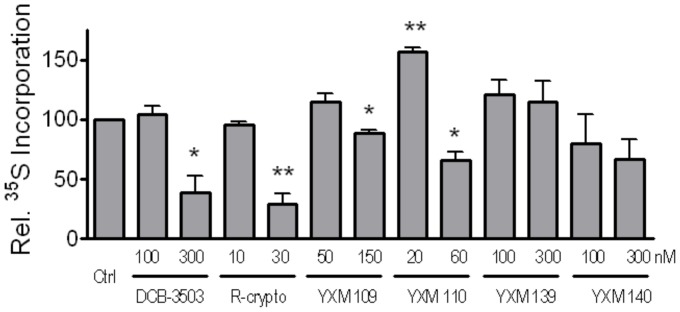
Effect of cryptopleurine analogs treatment for 4 hours on protein synthesis profile assessed by [^35^S]-methionine/cysteine incorporation in HepG2 cells. The incorporation of [^35^S]-methionine/cysteine was measure by scintillation counter, and presented as percentage to untreated control cells. (**, *p*<0.01; *, *p*<0.05).

We previously demonstrated that DCB-3503 inhibited protein synthesis at the elongation step of translation, which could be the basis of inhibiting cell growth and TNF/NF-κB pathway [8]. The effect of some cryptopleurine analogs on synthesis of cellular proteins was examined. Figure 4 showed that DCB-3503, Rac-cryptopleurine, YXM-109, 110, and 140 inhibited incorporation of [^35^S]-methionine/cysteine into newly synthesized proteins after treatment for 4 hours at their IC_50_ concentration. However, the treatment of YXM-139 did not show similar inhibition effect on protein synthesis under the same conditions.

**Figure 5:**
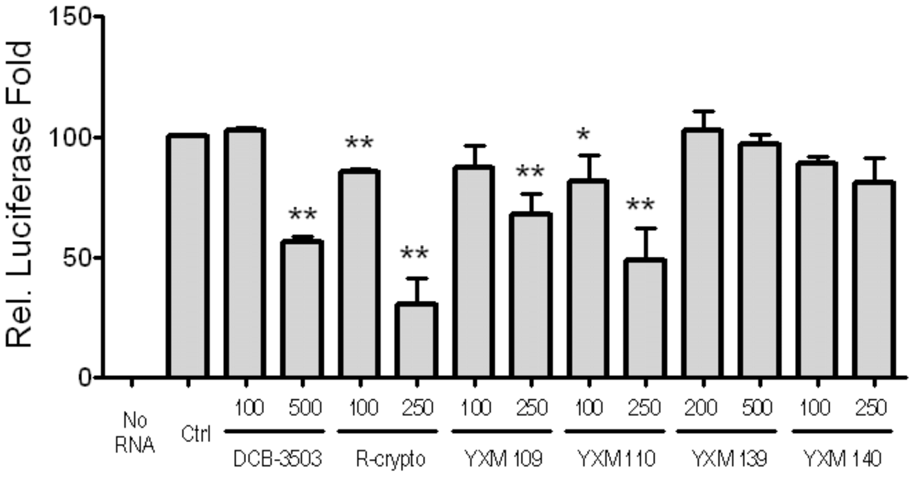
Cryptopleurine analogs inhibited luciferase translation in Retic lysate IVT system. Results were mean ± SD from three independent experiments. (**, *p*<0.01; *, *p*<0.05).

The effect of cryptopleurine analogs on protein translation was examined by capped luciferase mRNA with T3 promoter and poly (A) tail in Retic lysate *in vitro* translation system. DCB-3503 and Rac-cryptopleurine inhibited about 50% of translation of luciferase mRNA at 250 nM and 500 nM, respectively (Fig. 5). YXM-109 and 110 has significantly inhibitory effect on luciferase mRNA translation at 250 nM (*p*<0.05); however, YXM-139 and 140 did not inhibit translation under the same condition at 500 nM and 250 nM, respectively (Fig. 5). This suggested that modifications of the **E** ring not only altered cytotoxicity, but also changed their activity against protein translation.

### Conclusion

**Figure 6:**
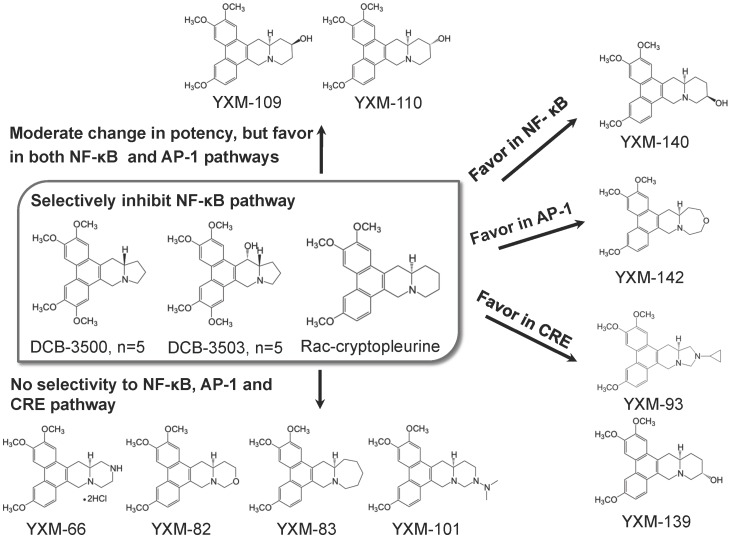
Schematic description of the SAR of cryptopleurine analogs with modification on the E-ring in HepG2 cell line.

Tylophorine analogs with introduction of different moieties (e.g. N or O) in the **E**-ring have less potency against the growth of HepG2 and Huh-7 cell lines, and some of those are no longer functional analogs. Introduction of the hydroxyl group into the **E**-ring altered the cytotoxicity of cryptopleurine ananlogs, and the chirality of the hydroxyl group is critical determination factor in the cytotoxicity. Cryptopleurine analogs with 6-member **E**-ring are more potent than 7-member **E**-ring analogs (Fig. 6 for results of HepG2 cells and Fig. S4 for results of HEK-293 cells). Together with results from our previous SAR studies [9], we propose the following order of potency in terms of NF-κB inhibition and cytotoxicity: six member **E**-ring with R14a-(R)-hydrogen>five member **E**-ring with R13a-(R)-hydrogen>five member **E**-ring with R13a-(S)-hydrogen>>six member **E**-ring with R14a-(R)-hydrogen. The results we obtained suggest that the **E** ring size may be a critical determination factor for the interaction of cryptopleurine analogs to their molecular targets, and molecular target of cryptopleurine analogs may not be identical. Changing structure of compound may not only be reflected in potency, but also determined the mode of action. Since the biochemical determinants of the primary target(s) of these compounds may vary, the selectivity against different signaling pathways could be cell type specific. This was demonstrated by the comparative study of HepG2 and HEK-293 cell lines. Their potency against HepG2 cell growth correlates well with the inhibitory activity against protein synthesis and TNF/NF-κB pathway. Tylophorine or Rac-cryptopleurine could be a good scaffold for synthesis of biological active compound with diverse action. We are in the process of evaluating the *in vivo* antitumor activity of selected cryptopleurine analogs with R15 hydroxyl group.

## Materials and Methods

### Materials

DCB-3503 (NSC-716802) was synthesized in Dr. D. C. Baker’s laboratory (The University of Tennessee, TN). Crytopleurine analogs were synthesized in Dr. K. H. Lee’s laboratory (University of North Carolina, NC). Cell culture media, fetal bovine serum (FBS) were purchased from Invitrogen (Carlsbad, CA). All chemicals except otherwise noted were purchased from Sigma-Aldrich (St. Louis, MO).

### Cell Lines and Growth Conditions

Cell lines were obtained from the American Type Culture Collection (ATCC). HepG2 cells were maintained in RPMI 1640 medium supplemented with 10% FBS. HepG2 stable cell lines harboring NF-κB, AP-1, CRE response elements in pGL4 vector (Promega) were maintained in the presence of 0.8 mg/ml G418. Huh-7 and HEK-293 cells were maintained in DMEM containing 4.5 g/l glucose supplemented with 10% FBS. All cell lines were maintained in a humidified incubator with an atmosphere of 95% air and 5% CO_2_ at 37°C.

### Cytotoxicity Assay

Ten thousands cells/well were plated in 24-well plates. After overnight incubation, cells were treated with drugs for 72 hours. Cells were fixed and stained with 0.5% methylene blue in 50% ethanol for 2 hours at room temperature, followed by washing with tap water to remove excess color. Plates were dried and then resuspended in 1% sarkosyl and incubate for 3 hours at room temperature. Cell growth was quantitated based on the amount of methylene blue adsorbed into cellular proteins measured by spectrophotometer (Molecular Devices) at 595 nm. IC_50_ was defined as the concentration of drug that inhibited cell growth by 50% after continuous drug exposure for 72 hours [1].

### Signaling Pathway Reporter Assay

HepG2 and HEK-293 cell lines stably harboring NF-κB, AP-1, and CRE response elements in pGL4.0 luciferase vector (Promega) were used for the signaling pathway reporter assay [1]. Cells were treated with 50 ng/ml TNF-α to stimulate NF-κB signaling pathway, 10 ng/ml TPA to stimulate AP-1, and 1 µM forskolin to stimulate CRE signaling pathway for 1 hour priory to addition of compounds for another 4 hours. Medium was removed at the end of the treatment, and cell extracts were prepared and luciferase activity was measured by Luciferase assay kit (Promega) according to the manufacturer’s instructions. IC_50_ was defined as the concentration of drug that inhibited stimulator-triggered luciferase reporter activation by 50% after continuous drug exposure for 4 hours.

### Western Blot Analysis

Western blot analysis was done using primary antibodies against Cox 2 (Cell Signaling Technology), iNOS (Abcam), cyclin D1 (Santa Cruz Biotechnology), and β-actin (Sigma-Aldrich) at optimal dilution [8].

### [^35^S]-amino Acid Mixture Incorporation Assay

The incorporation assay was done followed by the protocol described previously [8]. In brief, HepG2 cells treated with drugs were labeled with 50 µCi/ml [^35^S]-methionine/cysteine (PerkinElmer) for 30 minutes before harvest. Incorporation of [^35^S]-methionine/cysteine was determined by scintillation counter.

### *In vitro* Transcription

The detailed protocol for *in vitro* transcription was reported previously [8]. Luciferase encoding plasmid T3 luciferase was linearized by BamHI, and was used as the template for *in vitro* transcription. Capped luciferase mRNA was generated by mMESSAGE mMACHINE high yield capped RNA transcription kit containing T3 RNA polymerase (Ambion). The *in vitro* transcribed mRNAs were purified by MEGAclear kit (Ambion); and the integrity of mRNA was examined by Bioanalyzer (Agilent Technologies, Santa Clara, CA). The purified mRNAs were used for *in vitro* translation experiments.

### *In vitro* Translation

*In vitro* translation was performed by Retic Lysate IVT™ (Ambion) as described previously [8]. The *in vitro* translation mixtures containing 50 ng/µl T3 luciferase mRNA was incubated for 90 minutes at 30°C. Translation products of T3 luciferase were measured by luciferase assay.

### Statistical Analysis

Data were analyzed by ANOVA and the Bonferroni multiple comparision test by GraphPad Prism 5 software. The difference was considered to be statistically significant when *p*<0.05.

## Supporting Information

Figure S1 Dose-cell viability curves for DCB-3503 and cryptopleurine analogs in HepG2 (a) and Huh-7 (b) cell lines. Graphs were simplified by showing the mean value from three independent experiments.
(TIF)

Figure S2 Dose-luciferase activity curves for DCB-3503 and cryptopleurine analogs against NF-κB (a), AP-1 (b), and CRE (c) signaling pathways in HepG2 cell line. Graphs were simplified by showing the mean value from three independent experiments.
(TIF)

Figure S3 Dose-luciferase activity curves for DCB-3503 and cryptopleurine analogs against NF-κB (a), AP-1 (b), and CRE (c) signaling pathways in HEK-293 cell line. Graphs were simplified by showing the mean value from three independent experiments.
(TIF)

Figure S4 **Schematic description of the SAR of cryptopleurine analogs with modification on the E-ring in HEK-293 cell line.**
(TIFF)
