# Supplementary figures and images for: Cryptopleurine Analogs with Modification of E Ring Exhibit Different Mechanism to Rac-Cryptopleurine and Tylophorine
Source: PLoS One. 2012 Dec 10;7(12):e51138. doi: 10.1371/journal.pone.0051138 (PMC3519526; doi:10.1371/journal.pone.0051138)

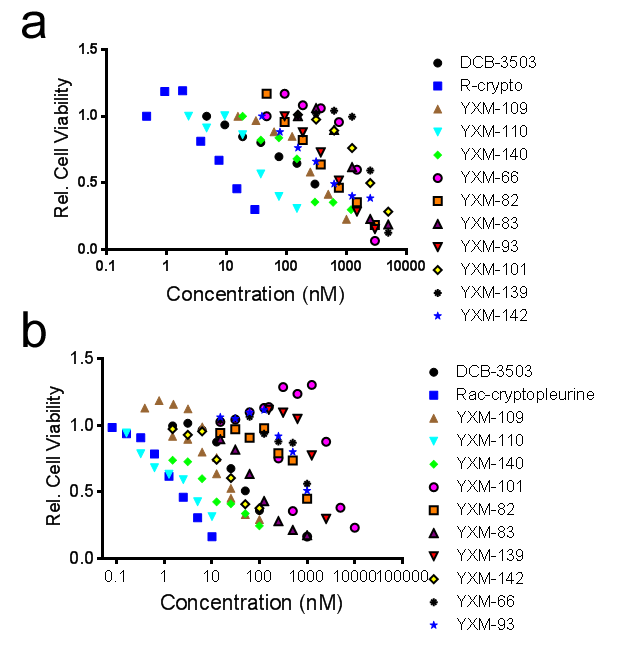

Supplement: Figure S1 — Dose-cell viability curves for DCB-3503 and cryptopleurine analogs in HepG2 (a) and Huh-7 (b) cell lines. Graphs were simplified by showing the mean value from three independent experiments. (TIF) [file pone.0051138.s001.tif]

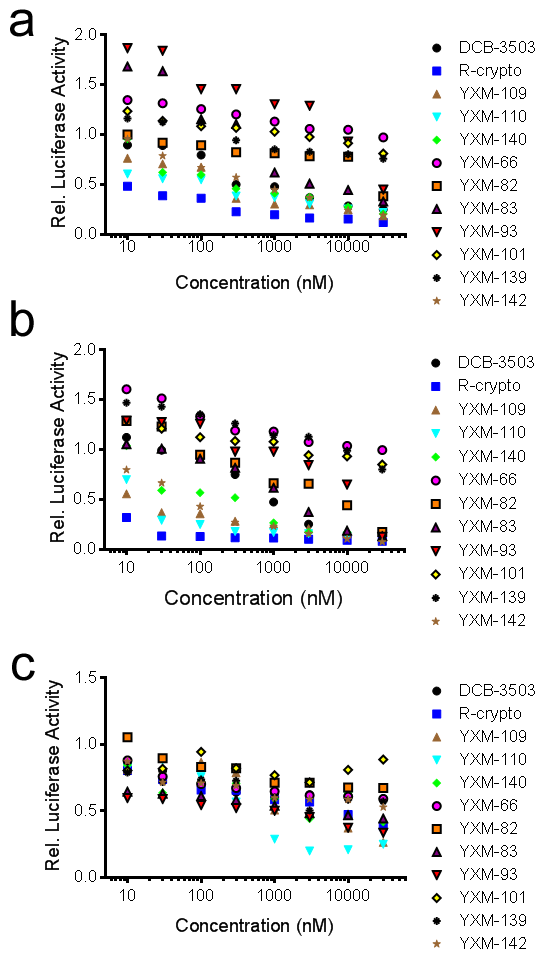

Supplement: Figure S2 — Dose-luciferase activity curves for DCB-3503 and cryptopleurine analogs against NF-κB (a), AP-1 (b), and CRE (c) signaling pathways in HepG2 cell line. Graphs were simplified by showing the mean value from three independent experiments. (TIF) [file pone.0051138.s002.tif]

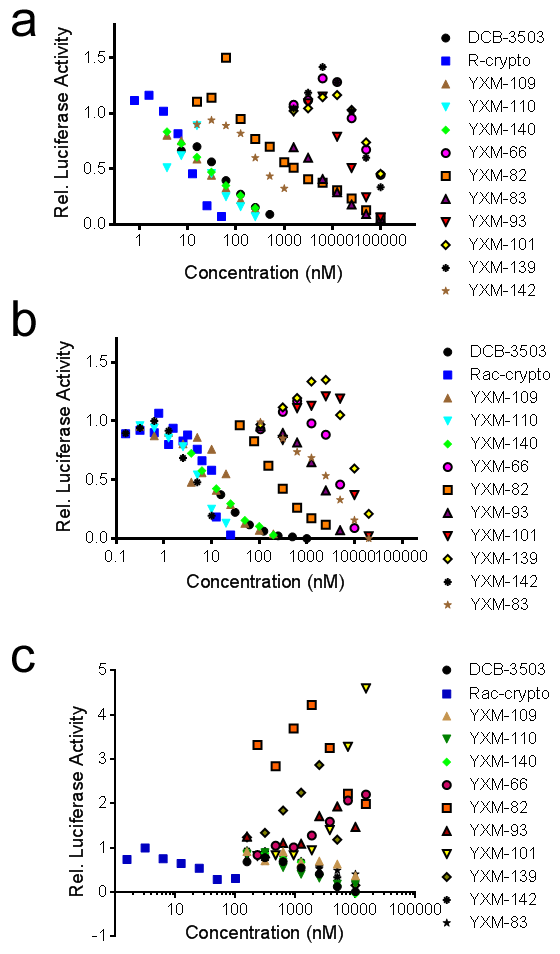

Supplement: Figure S3 — Dose-luciferase activity curves for DCB-3503 and cryptopleurine analogs against NF-κB (a), AP-1 (b), and CRE (c) signaling pathways in HEK-293 cell line. Graphs were simplified by showing the mean value from three independent experiments. (TIF) [file pone.0051138.s003.tif]

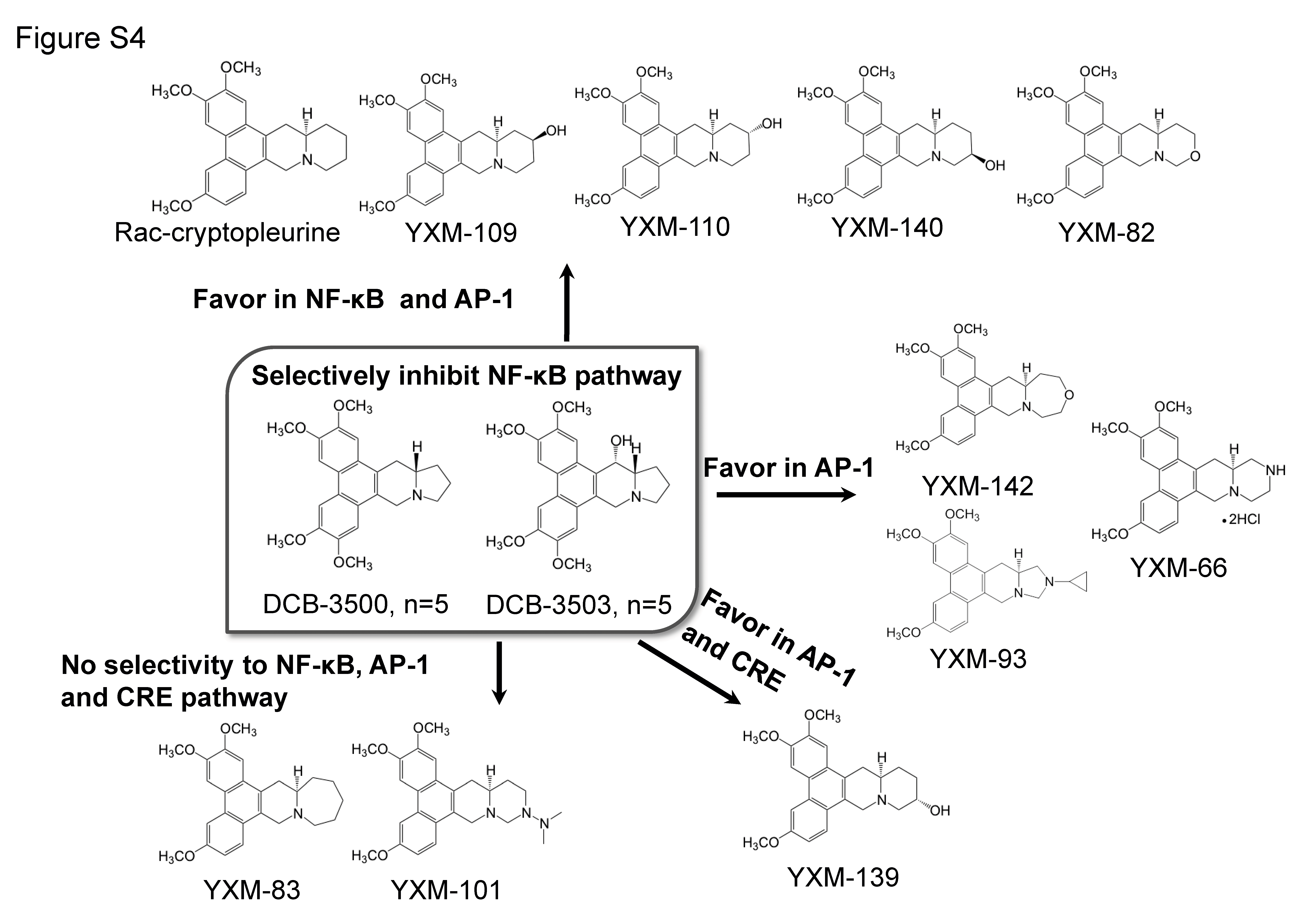

Supplement: Figure S4 — Schematic description of the SAR of cryptopleurine analogs with modification on the E-ring in HEK-293 cell line. (TIFF) [file pone.0051138.s004.tiff]
